# Supplementary material for: Identification of diagnostic candidates in Mendelian disorders using an RNA sequencing-centric approach
Source: Genome Med. 2024 Sep 9;16:110. doi: 10.1186/s13073-024-01381-w (PMC11382415; doi:10.1186/s13073-024-01381-w)
Supplement: Supplementary file 1 — Additional file 1: Supplemental methods and results. Additional information on sample collection, RNA extraction, RT-PCR and Sanger sequencing methods. Overview of aberrant splicing events identified using LeafCutterMD, MAJIQ, FRASER2 and rMATS-turbo and normalisation of data with OUTRIDER and FRASER2. [file 13073_2024_1381_MOESM1_ESM.docx]

## Supplemental methods

## Sample collection and RNA extraction

RNA was extracted from 87 blood samples collected in PAXgene blood RNA tubes using the PAXgene blood RNA Kit (PreAnalytiX, Switzerland). Quantification was performed by NanoDrop spectrophotometer and Qubit fluorometer (Thermo, MA) and RNA integrity number assessed using an Agilent 2100 Bioanalyzer (Agilent, CA).

## RT-PCR and Sanger sequencing

Primers for the variant of interest were designed to span at least three exons and where possible, up to seven exons. The cDNA was synthesised using High-Capacity cDNA Reverse Transcription Kit (Thermo Fisher Scientific, USA). PCR was performed using GoTaq G2 DNA polymerase kit (Promega, USA). The PCR products were analysed in a 1% agarose gel prepared with Nancy-520 DNA gel stain (Sigma, USA). Subsequently, the PCR products were purified using the GeneJET PCR Purification Kit (Thermo Fisher Scientific, USA) and bidirectional Sanger sequencing was carried out by SourceBioscience. PCR experiments were repeated for reproducibility.

**Supplemental Results**

**Identification of aberrant splicing events**

LeafCutterMD identified an average of 1,134 (593-3,345) significant event (raw p-values ≤ 0.05) with an effect size greater than 0.2 or less than -0.2. The MAJIQ voila module DeltaPSI identified an average 1,380 (781-3,699) significant events per sample. FRASER identified an average of 1,859 (615-4,164) significant events. While rMATS-turbo identified an average of 3,578 (1,477-115,522) significant events per sample with an inclusion level greater than 0.2 or less than -0.2. Our first approach using filtered VCF files to extract AS events within 25 base pairs of a variant reduced the mean number of events per proband ~300 fold (rMATS-turbo: 12 (0-69); MAJIQ: 9 (0-40), LeafCutterMD: 3 (0-14) and FRASER: 3 (0-100).


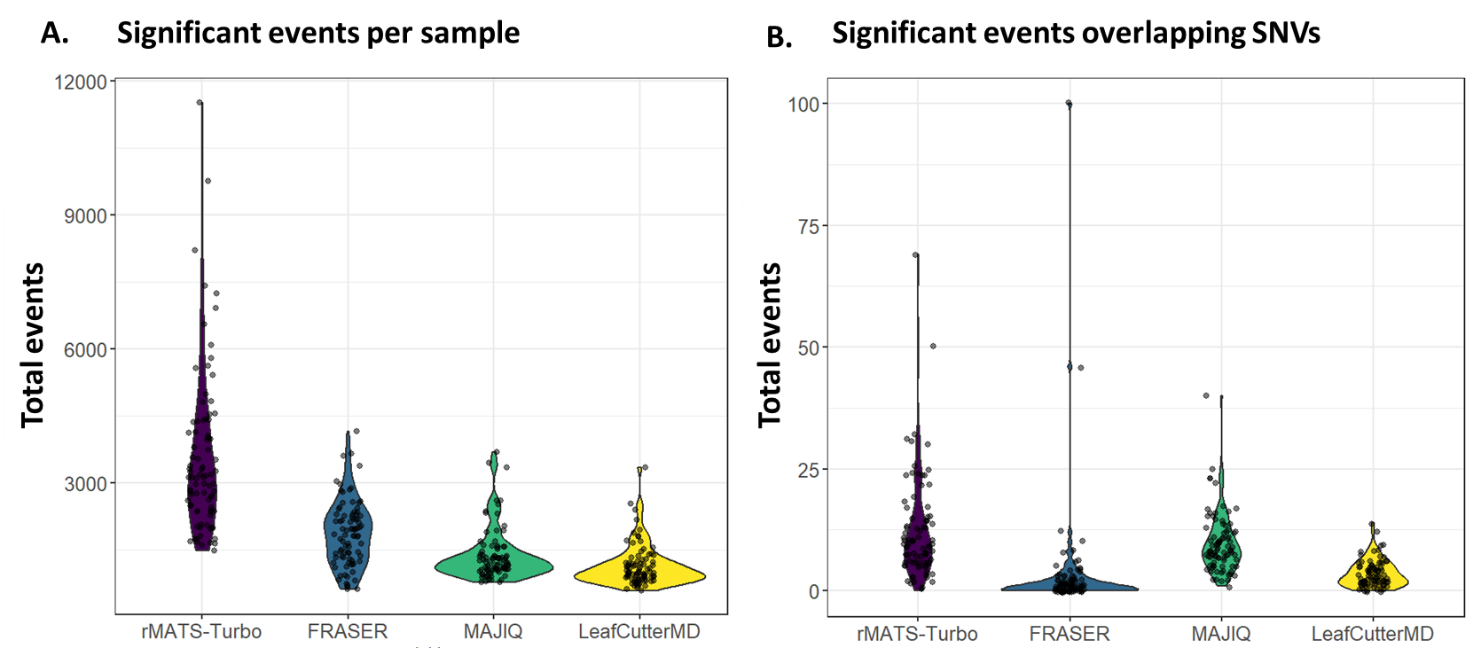


***Figure SR1****. Overview of events identified by the four splicing tools.* ***A)*** *Distribution of significant events (p-value < 0.05) with an effect size greater than 0.2 or less than -0.2 across all three splicing tools. FRASER, LeaftCutterMD and MAJIQ have a similar distribution while rMATS-turbo identifies around three times as many significant events.* ***B)*** *Distribution of significant events (p-value < 0.05) with an effect size greater than 0.2 or less than -0.2 that overlap an SNV. After filtering events using variant calling data, the number of events across all three tools was reduced drastically and the number of events identified by rMATS-turbo were in line with those identified by the other three tools.*

**Normalisation of data within OUTRIDER and FRASER**

Both OUTRIDER and FRASER provide options to control for confounders. The data is visualised in a heatmap as this can show if samples cluster together before and after normalisation. OUTRIDER does a better job of correcting for potential confounders than FRASER as seen in the heatmaps below.

**
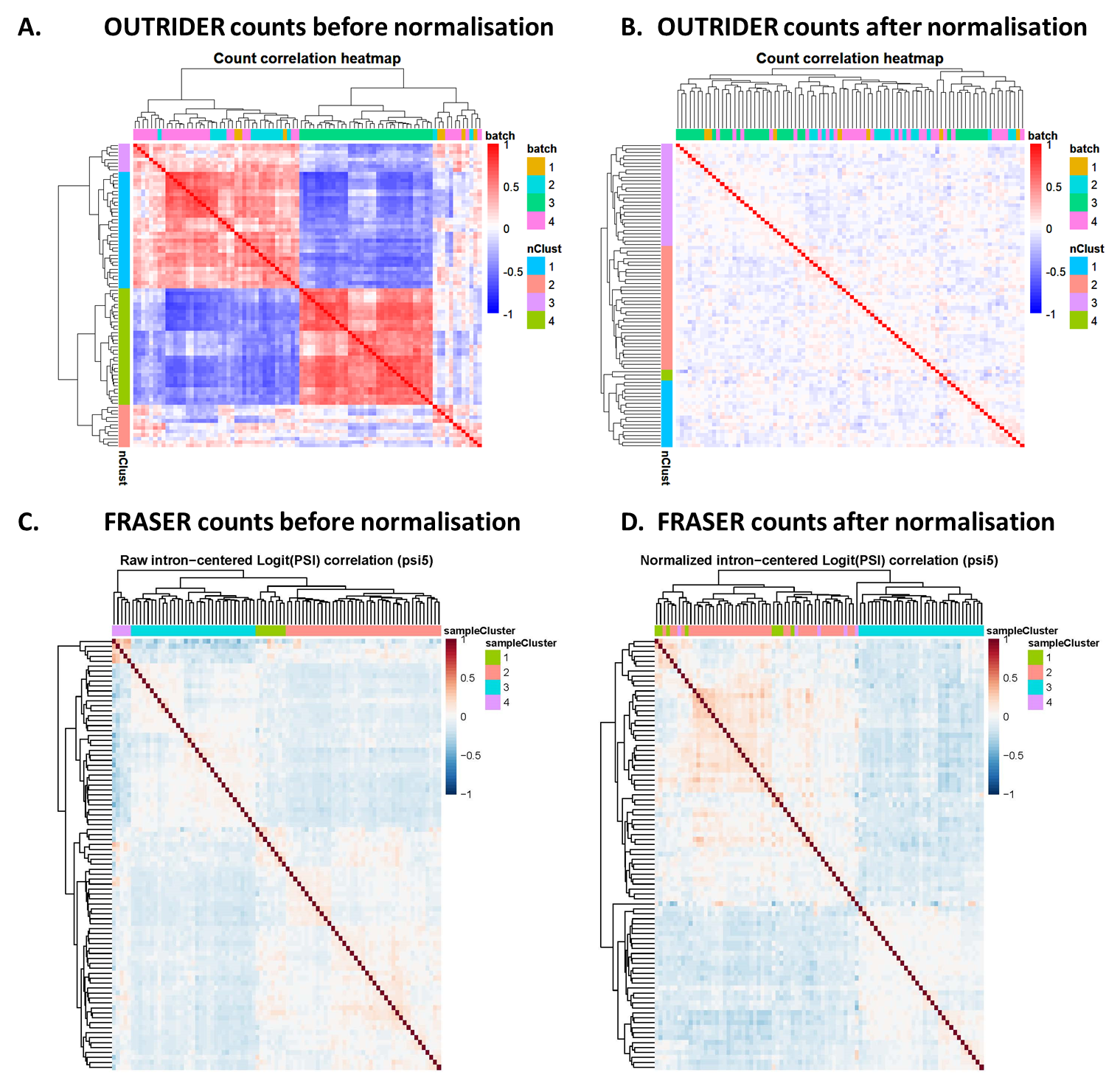
 *Figure SR2****. Effect of count normalisation in OUTRIDER (****A*** *and* ***B****) and FRASER (****C*** *and* ***D****). Plots were generated using the plotCountCorHeatmap() function.*
