# Supplementary material for: Identification of diagnostic candidates in Mendelian disorders using an RNA sequencing-centric approach
Source: Genome Med. 2024 Sep 9;16:110. doi: 10.1186/s13073-024-01381-w (PMC11382415; doi:10.1186/s13073-024-01381-w)
Supplement: Supplementary file 3 — Additional file 3: Supplemental figures. Fig. S1 Overview of RNA sequencing data. Fig. S2 Median transcript per million (TPM) values in genes with a variant of uncertain significance (VUS) across the four batches. Fig. S3 Effects of globin depletion on gene expression. Fig. S4 Intron retention caused by intronic variant in UBR4. Fig. S5 Gene rank plots for DKC1 and P3H1. Fig. S6 GTEx blood TPM values across selected different gene panels available via the UK Genomic Medicine Service. [file 13073_2024_1381_MOESM3_ESM.pdf]

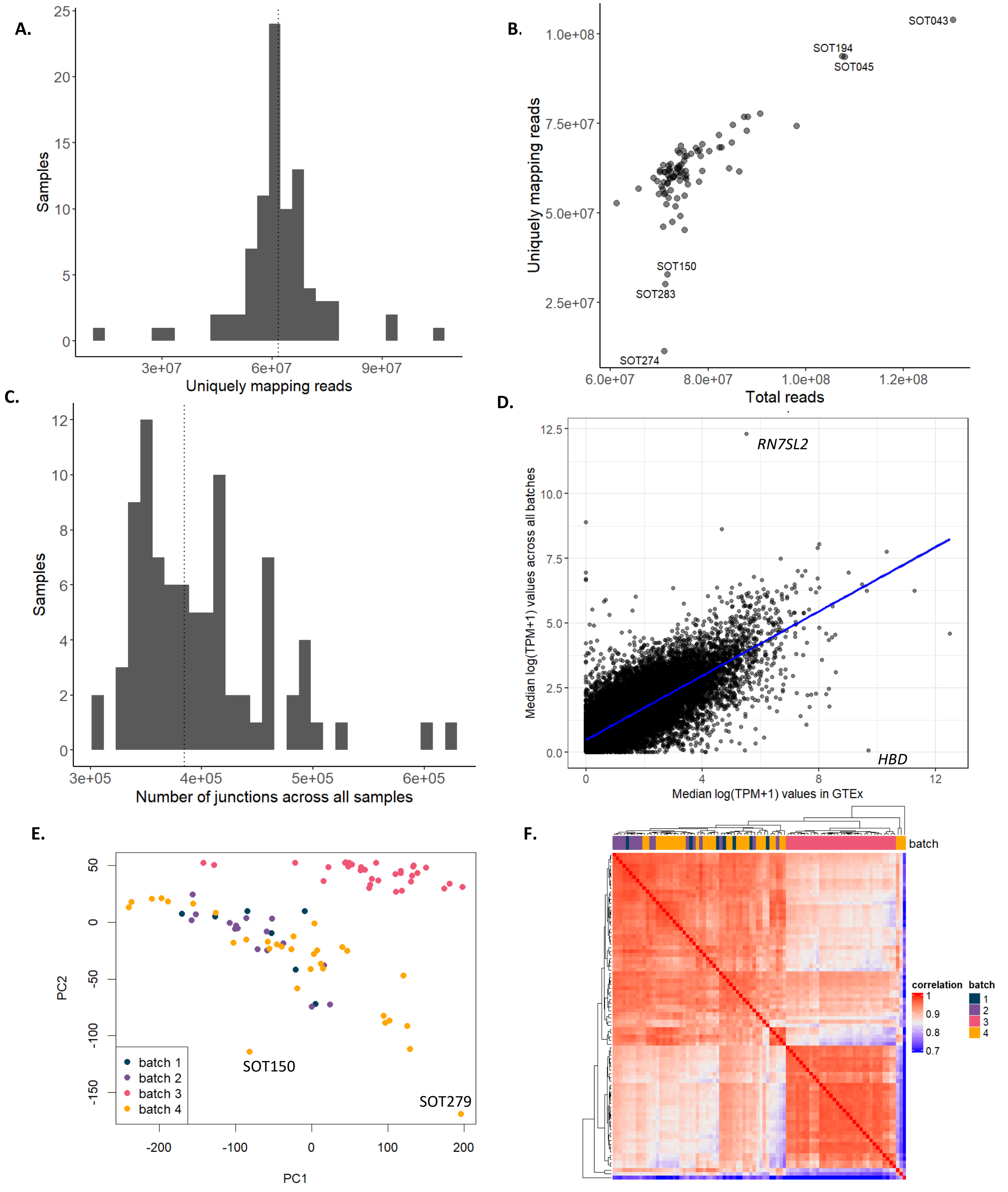

Figure S1. Overview of RNA sequencing data. **A)** Distribution of total number of uniquely mapping reads across all samples. **B)** Total number of reads vs number of uniquely mapped reads. Samples SOT274, SOT283 and SOT150 had fewer uniquely mapping reads than the rest of the cohort. **C)** Distribution of the total number of junctions identified across all samples (number extracted from SJ.out.tab from STAR). **D)** Correlation of median TPM values within the cohort versus median TPM in GTEx. Values are shown on a log scale. **E)** Principal component (PC) analysis generated using log scaled counts per million in R. As expected, the first two PCs show batch three as a separate cluster and no clear separation between other batches. **F)** Correlation of transcriptomic profiles across samples. Like the PC analysis this was generated using the log scaled counts per million using in R. Here batch three also clusters together highlighting the differences between targeting methods.

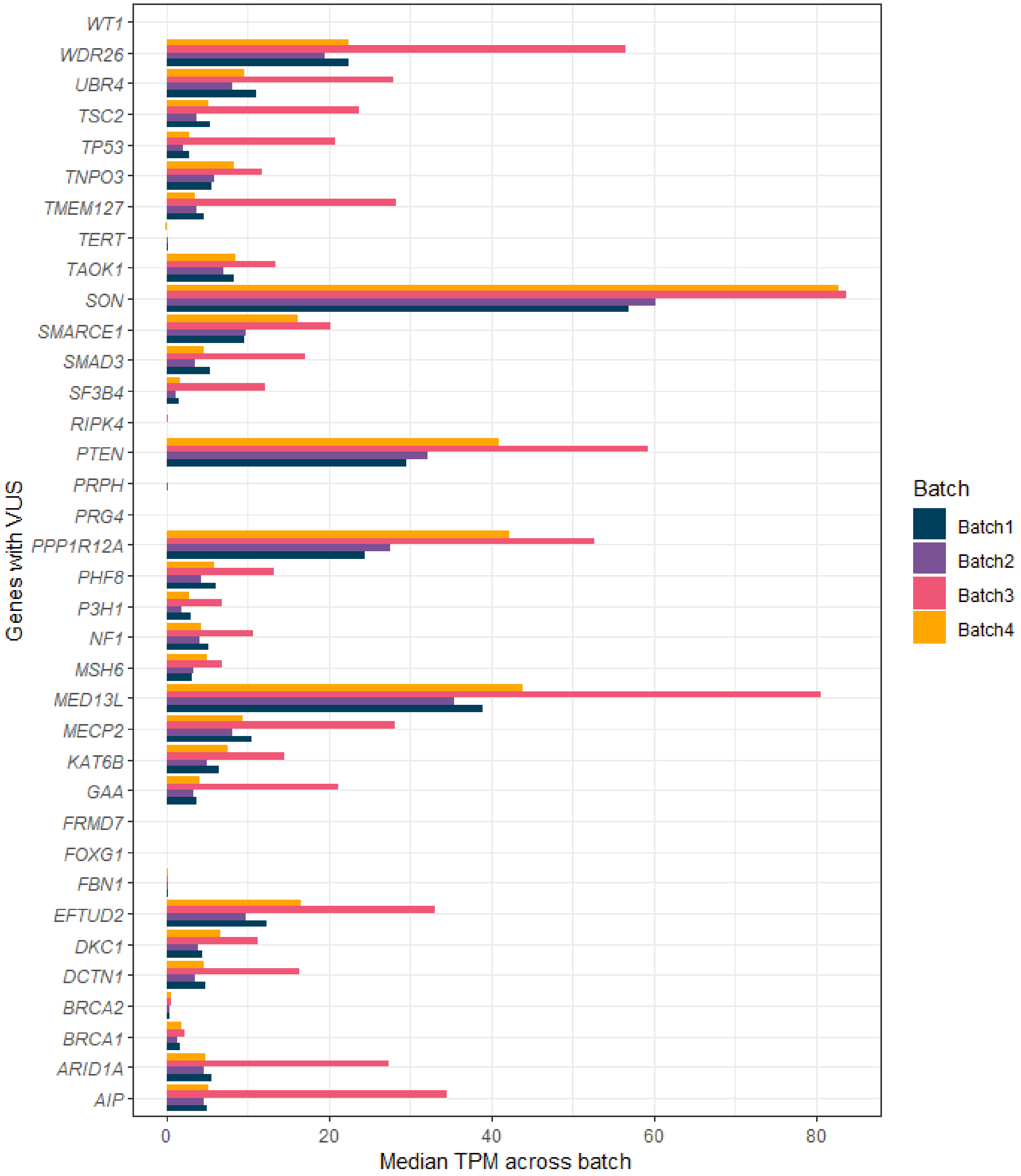

Figure S2. Median transcript per million (TPM) values in genes with a variant of uncertain significance (VUS) across the four batches. Batch 3 is shown in pink, which shows that lack of globin depletion did not affect coverage in genes of interest.

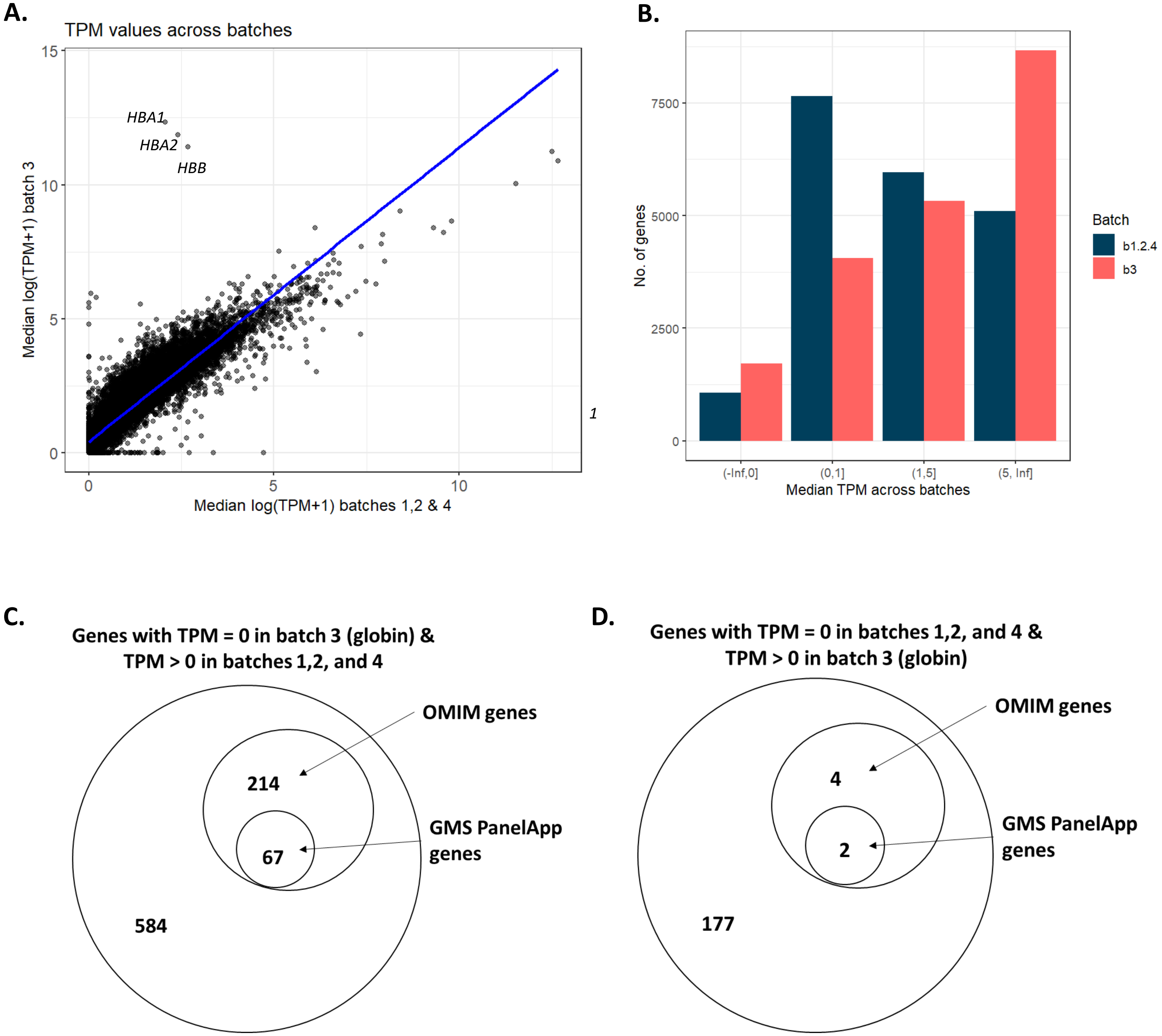

Figure S3. Effects of globin depletion on gene expression. A**)** Correlation of median TPM values across batch 3 versus the other three batches (1,2, and 4). Values are shown on a log scale. Haemoglobin genes *HBB, HBA1* and *HBA2* show much higher expression in batch 3 as expected. Overall, the lack of globin depletion does not seem to have a large effect on the transcriptomic profiles **B)** Distribution of TPM values across four discrete bins. When we group gene expression by bins, we see the largest difference in the genes with 0 > TPM > 1 as well as TPM > 5. **C)** Breakdown of genes with TPM of 0 in batch 3 & TPM > 0 in other batches. **D)** Breakdown of genes with TPM > 0 in batch 3 & TPM = 0 in other batches.

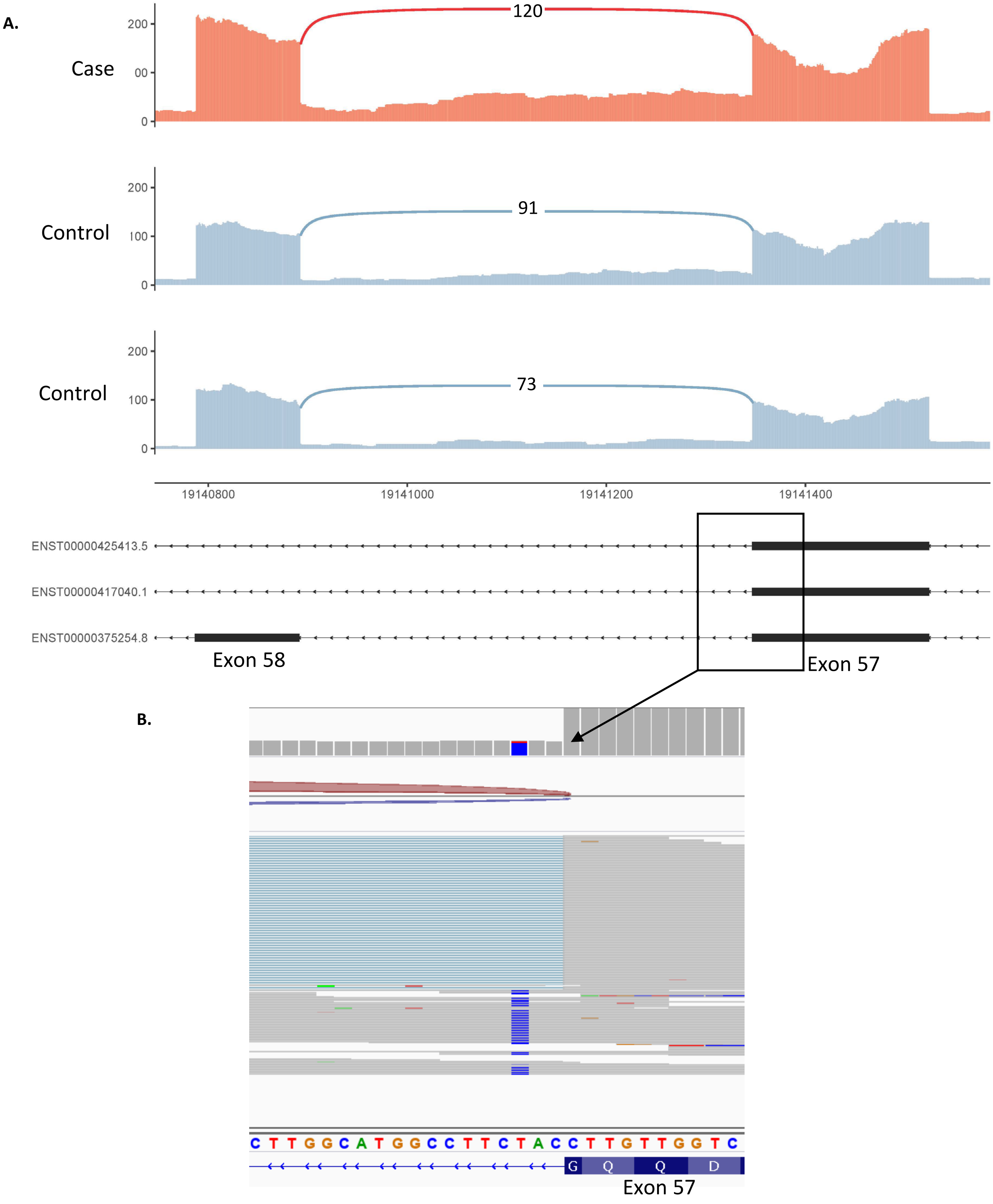

Figure S4. Intron retention caused by intronic variant in *UBR4*. **A)** Sashimi plot of the proband and two controls of the *UBR4* region of interest. For the proband only (red track), there is an increase in the number of intronic reads compared to the controls. **B)** IGV screenshot of coverage across region of interest. The variant (blue) is present in 46 reads.

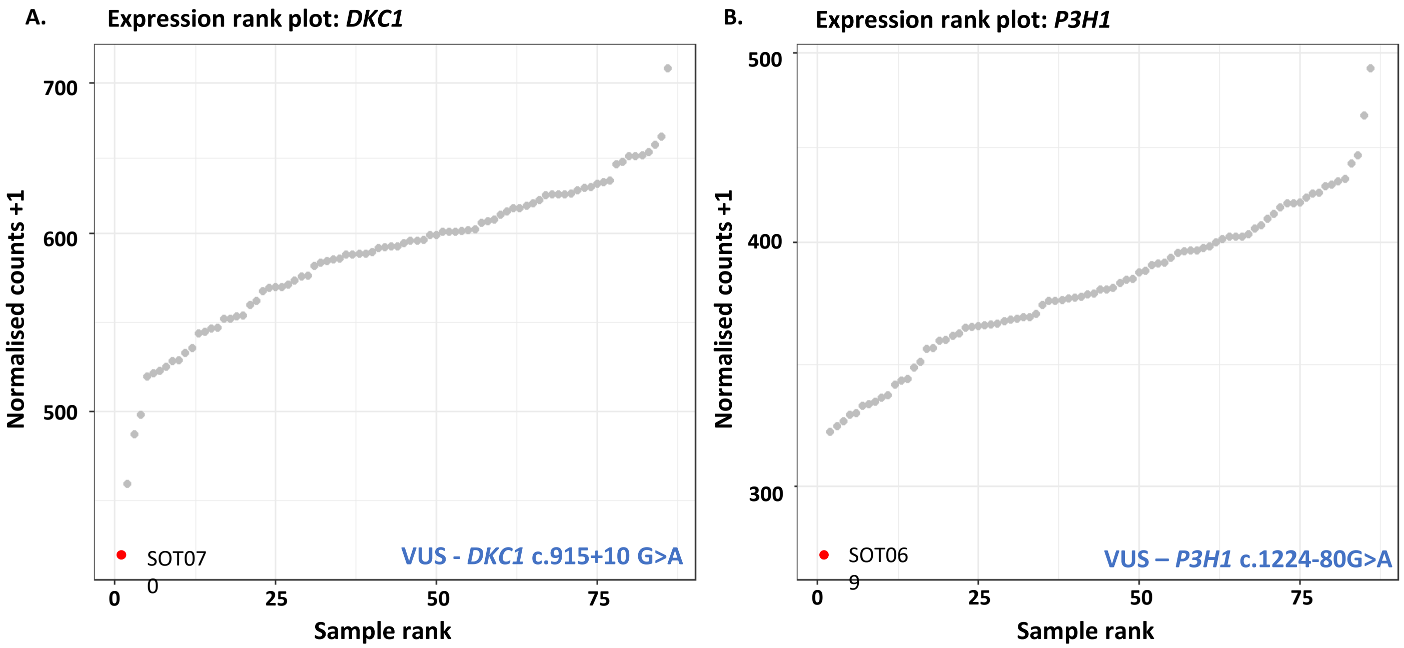

Figure S5. Gene rank plots for *DKC1* and *P3H1*. **A)** Normalised expression of *DKC1* gene ranked across all samples. Red dot indicates patient with *DKC1*.c915+10G>A variant. **B)** Normalised expression of *P3H1* gene ranked across all samples. Red dot indicates patient with *P3H1*:c.1224-80G>A variant. Rank 1 indicates sample had the lowest normalised expression across the entire cohort.

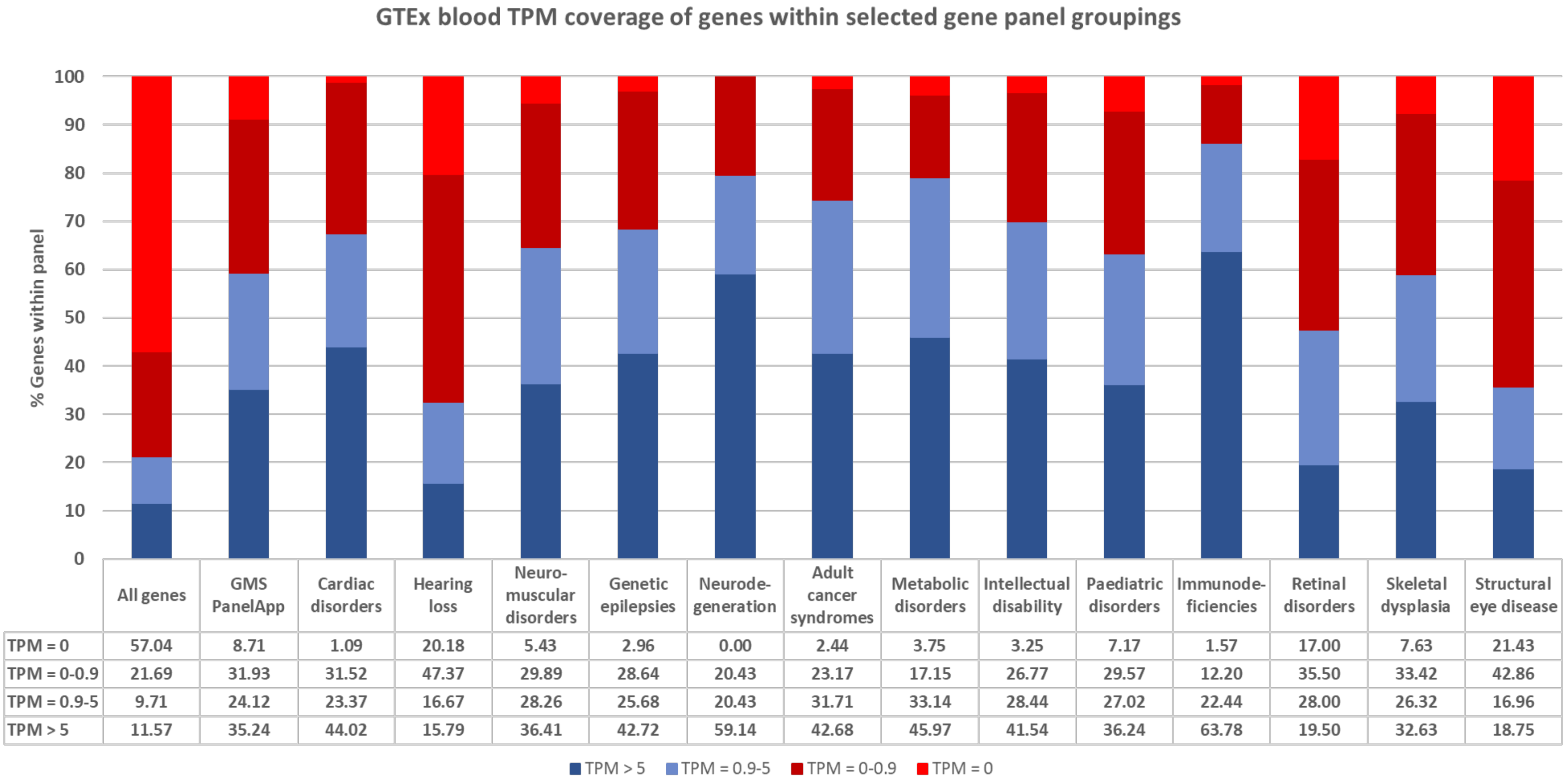

	All genes	GMS PanelApp	Cardiac disorders	Hearing loss	Neuro-muscular disorders	Genetic epilepsies	Neurode-generation	Adult cancer syndromes	Metabolic disorders	Intellectual disability	Paediatric disorders	Immunode-ficiencies	Retinal disorders	Skeletal dysplasia	Structural eye disease
TPM = 0	57.04	8.71	1.09	20.18	5.43	2.96	0.00	2.44	3.75	3.25	7.17	1.57	17.00	7.63	21.43
TPM = 0-0.9	21.69	31.93	31.52	47.37	29.89	28.64	20.43	23.17	17.15	26.77	29.57	12.20	35.50	33.42	42.86
TPM = 0.9-5	9.71	24.12	23.37	16.67	28.26	25.68	20.43	31.71	33.14	28.44	27.02	22.44	28.00	26.32	16.96
TPM > 5	11.57	35.24	44.02	15.79	36.41	42.72	59.14	42.68	45.97	41.54	36.24	63.78	19.50	32.63	18.75

■ TPM > 5 ■ TPM = 0.9-5 ■ TPM = 0-0.9 ■ TPM = 0

Figure S6. GTEx blood TPM values across selected different gene panels available via the UK Genomic Medicine Service. RNA-seq data allowed assessment of splicing in genes with at least TPM ≥ 5 (dark blue) and in some cases where TPM was less than 5 and ≥ 0.9 (light blue). Genes with TPM less than 0.9 and ≥ 0 (dark red) may still prove testable in terms of RT-PCR splicing analysis of VUSs.
